# Dynamic Hebbian Cross-Correlation Learning Resolves the Spike Timing Dependent Plasticity Conundrum

**DOI:** 10.3389/fncom.2017.00119

**Published:** 2018-01-11

**Authors:** Tjeerd V. olde Scheper, Rhiannon M. Meredith, Huibert D. Mansvelder, Jaap van Pelt, Arjen van Ooyen

**Affiliations:** ^1^Department of Integrative Neurophysiology, Center for Neurogenomics and Cognitive Research, Vrije Universiteit Amsterdam, Amsterdam, Netherlands; ^2^Department of Computing and Communication Technologies, Faculty of Technology, Design and Environment, Oxford Brookes University, Oxford, United Kingdom

**Keywords:** spike timing-dependent plasticity, dynamic systems, synaptic stability, network stability, computational modeling

## Abstract

Spike Timing-Dependent Plasticity has been found to assume many different forms. The classic STDP curve, with one potentiating and one depressing window, is only one of many possible curves that describe synaptic learning using the STDP mechanism. It has been shown experimentally that STDP curves may contain multiple LTP and LTD windows of variable width, and even inverted windows. The underlying STDP mechanism that is capable of producing such an extensive, and apparently incompatible, range of learning curves is still under investigation. In this paper, it is shown that STDP originates from a combination of two dynamic Hebbian cross-correlations of local activity at the synapse. The correlation of the presynaptic activity with the local postsynaptic activity is a robust and reliable indicator of the discrepancy between the presynaptic neuron and the postsynaptic neuron's activity. The second correlation is between the local postsynaptic activity with dendritic activity which is a good indicator of matching local synaptic and dendritic activity. We show that this simple time-independent learning rule can give rise to many forms of the STDP learning curve. The rule regulates synaptic strength without the need for spike matching or other supervisory learning mechanisms. Local differences in dendritic activity at the synapse greatly affect the cross-correlation difference which determines the relative contributions of different neural activity sources. Dendritic activity due to nearby synapses, action potentials, both forward and back-propagating, as well as inhibitory synapses will dynamically modify the local activity at the synapse, and the resulting STDP learning rule. The dynamic Hebbian learning rule ensures furthermore, that the resulting synaptic strength is dynamically stable, and that interactions between synapses do not result in local instabilities. The rule clearly demonstrates that synapses function as independent localized computational entities, each contributing to the global activity, not in a simply linear fashion, but in a manner that is appropriate to achieve local and global stability of the neuron and the entire dendritic structure.

## 1. Introduction

Spike Timing-Dependent Plasticity (STDP) (Bi and Poo, 1998) is regarded as a major progression toward understanding the problem of how learning is achieved in biological neuronal networks. Because STDP gives a synapse-specific, non-tetanic means of regulating synaptic plasticity, learning in biological neuronal networks seems reduced to a simple time dependent rule (Song et al., 2000). As is so often the case, biological experimental results have shown that STDP appears to be more complex than initially assumed. It has been shown that STDP takes different forms during development (Wittenberg and Wang, 2006), and that there are variations within brain regions and species (Buchanan and Mellor, 2010; Testa-Silva et al., 2010). It is frequency, and location dependent (Sjöström et al., 2001; Froemke et al., 2010). Inhibition appears to invert the STDP curve, which seemingly contradicts the Hebbian nature of STDP (Lamsa et al., 2010). Even the existence of STDP as a valid learning mechanism has been questioned (Lisman and Spruston, 2010). Different reviews of STDP have emphasized the possible variations and forms of STDP (Morrison et al., 2008; Sjöström et al., 2008; Buchanan and Mellor, 2010), but so far no satisfactory explanation has been proposed how these different forms may arise.

We have defined a novel dynamic framework which can address the STDP conundrum by considering the synapse as an independent Hebbian entity. It relates the synaptic strength to the presynaptic and postsynaptic activity as the result of localized independent postsynaptic dynamics, which is founded on experimental results (Makino and Malinow, 2011). The localized dynamics are formed by the presynaptic input, the postsynaptic response to the input and the presence of postsynaptic activity due to other processes, such as other synapses and action potentials. This Dynamic Hebbian Learning model (dynHebb) demonstrates the cardinal role of local dynamics to synaptic learning beyond global neural behavior. The dynHebb model shows that both Hebbian and anti-Hebbian learning depend on several contributing factors, namely spike timing, the amount of postsynaptic activity, and adaptation by postsynaptic action potentials as well as inhibitory input. The mechanism of STDP learning takes on several dimensions of complexity simply due to local dynamic interactions, and the relevance of specific activities and inhibition to the input. Synapses can learn and contribute to the global neuronal behavior but are also subject to local rules that determine the effective individual synaptic strength independently.

The dynHebb model is based on the causal cross-correlation between pre- and postsynaptic activity as expressed by a phenomenological activity measure. The model is based on the following three guiding principles. Firstly, a distinction is made between local postsynaptic activity at the synapse (henceforth denoted as *P*_post_), directly resulting from the presynaptic activity (denoted as *P*_pre_), and local dendritic activity resulting from all activity sources in the local dendrite (denoted as *P*_*d*_). Secondly, the cross-correlation between presynaptic *P*_pre_ and local postsynaptic activity *P*_post_ determines the potential for synaptic efficacy. This cross-correlation reflects synaptic depression due to presynaptic activity if little or none local postsynaptic activity is present. Thirdly, the cross-correlation of the local postsynaptic activity of the synapse *P*_post_ with the dendritic activity *P*_*d*_, which has been shown to be essential for STDP (Kampa et al., 2007). The dendritic activity is formed by all contributing factors that form this activity, such as Back-propagating Action Potentials (BAPs), and activities of other nearby synapses (including the local postsynaptic activity). The interaction between local postsynaptic activities are directly responsible for different forms of STDP learning (Lamsa et al., 2010). In the dynHebb model, the three principles are contributing parts of an autonomous (time-independent) learning rule from which time dependent STDP learning emerges. Due to its dynamic nature, the autonomous learning rule responds in a simple feed-forward manner to the synaptic input, eliminating the need to go back in time and adjust synaptic strengths such as is required for the standard STDP model (Song et al., 2000). The relation between the dynamics of a single synapse and the postsynaptic dynamics in the dendrite becomes apparent by different emerging learning behavior. The presence of BAPs and synaptic inhibition changes the shape of the single STDP learning rule. The dendritic activity formed by other sources than the synapse makes a significant contribution to the learning rule, and can control the synaptic dynamics completely. Because the model also describes the relation between the presynaptic activity and the local postsynaptic activity caused by the synapse, as well as other dendritic activity due to nearby competing synapses, the mechanism enables the synapse to respond to input as well as compete with other synapses and tune itself to the global neuronal activity.

## 2. Methods

In this section, we describe the underlying model of the Dynamic Hebbian Learning Rule, and its basic properties. In the Results section 3, we will elaborate in detail on the consequences of the methods, in particular, the dynamic interactions that permit the emergence of the learning rules.

### 2.1. Dynamic hebbian learning rule

We construct a specific learning rule (dynHebb) based on the comparison between the local postsynaptic activity *P*_post_ of a given synapse directly resulting from presynaptic activity *P*_pre_, and the global postsynaptic activity resulting from the combination of all local sources in the local dendrite *P*_*d*_ (including the *P*_post_ of a given synapse). The negated causal cross-correlation of presynaptic and postsynaptic activity, *P*_pre_ and *P*_post_, is scaled by the dendritic activity *P*_*d*_ which ensures stability of the synaptic strength. This represents the direct effectiveness of the synapse by relating the presynaptic activity with the filtered postsynaptic response it has generated. Hence, the negated causal cross-correlation causes synaptic depression and this can only be overcome by the potentiation due to a robust postsynaptic response. This response is based on the postsynaptic activity generated by the synapse and the cross-correlation of this activity with the dendritic activity. If sufficiently large postsynaptic activity is present when the synapse is active (i.e., when the cross-correlation terms are not zero), the synapse becomes potentiating by overcoming the initial synaptic depression. To avoid unnecessary confusion regarding the biological interpretation of the activity term, it suffices to consider it an abstraction similarly to the postsynaptic potential in functionality.

### 2.2. Synaptic activity model

The change in synaptic weight *W* in the Synaptic Activity Model (SAM) is described by the following equations

(1) $$F_{\text{dyn}}^{i}(t)=\frac{aP_{\text{pre}}P_{\text{post}}}{\left(1+dP_{d}\right)}+bP_{\text{post}}+cP_{\text{post}}P_{d}$$

(2) $$\frac{dW_{i}(t)}{dt}=F_{\text{dyn}}^{i}(t)-eW_{i}(t)$$

where *W*_*i*_(*t*) is the synaptic weight of synapse *i*, $F_{\text{dyn}}^{i}$ the dynamic cross-correlation learning rule for the same synapse, *P*_pre_ is the presynaptic activity, *P*_post_ the resulting local postsynaptic activity of the synapse and *P*_*d*_ the local dendritic activity.

To encapsulate the contribution of the localized memory that each synapse contributes to the global dynamics, the second term in Equation (1) represents the linear local response of the postsynaptic activity. This term may be considered to provide a linearly scaled direct response to the input, allowing local activity to remain after input has died away. To provide suitable functions for the cross-correlation terms, the synaptic activity is formed by a descriptive decay function, based on the alpha function (Koch, 1999) commonly used to model neural potentials. Here, the activity function uses a set of two second order differential equations, which allows adaptive response to local activity patterns instead (olde Scheper et al., 2012). Each of the *P*_pre_, *P*_post_, and *P*_*d*_ terms are described by *P*_*x*_ in

(3) $$\frac{d\;P_{x}}{d\;t}=\alpha \;Q^{2}+\beta \;{P_{x}}^{2}$$

(4) $$\frac{d\;Q}{d\;t}=\phi \;Q^{2}+W\;\gamma \;I$$

where α = 1, β = −1, ϕ is in the range [−2, 0〉, γ ≥ 1, *W* is the synaptic strength in case of the *P*_pre_, *I* is the input, *Q* is the input variable and *P* represents the synaptic activity.

The choice of parameter values *a* = −1, *b* = 0.3, *c* = 0.1 and *e* = −0.001 is not critical, but reflects the relative contribution of the three terms. Where appropriate, these can be modified to study specific dynamics, similarly as the α decay term in the alpha-function. In particular, the parameters *a* and *c* and the decay parameter *e* can be modified to suit specific learning rates. The advantages of the SAM approach are that the autonomous model does not require specific detection of a neural spike and can be modified with parameter *W* in a feed-forward manner. Contributing postsynaptic activities can also be added and scaled, if necessary (olde Scheper et al., 2012). The SAM is used to connect suitable spiking membrane models, such as the Hindmarsh-Rose model (Hindmarsh and Rose, 1984) or the Morris-Lecar model (Izhikevich, 2004), by providing scaled input *I* to the pre-synaptic activity *P*_pre_ (Equation 4) for which the weight is unity. Then the post-synaptic local activity *P*_post_ is determined based on the same spiking model but weighted *W*_*i*_, also according to Equation (4). In this paper, we used the Morris-Lecar model, which is described by

(5) $$\begin{array}{l} C\frac{d\;V}{d\;t}=I_{s}-g_{L}(V-V_{L})-g_{ca}m_{\infty }(V)(V-V_{ca})\\ \;-g_{K}n(V-V_{K}) \end{array}$$

(6) $$\begin{array}{l} \;\frac{d\;n}{d\;t}=\lambda (V)(n_{\infty }(V)-n) \end{array}$$

where

(7) $$m_{\infty }=\frac{1}{2}\left\{1+\tanh\left(\frac{\left(V-V_{1}\right)}{V_{2}}\right)\right\}$$

(8) $$n_{\infty }=\frac{1}{2}\left\{1+\tanh\left(\frac{\left(V-V_{3}\right)}{V_{4}}\right)\right\}$$

(9) $$\lambda (V)=\bar{\lambda }\cosh\left(\frac{\left(V-V_{3}\right)}{2V_{4}}\right)$$

with *C* = 20 μF/cm^2^, $g_{L}=2\text{ mS}/\text{c}{\text{m}}^{2}$,*V*_*L*_ = −50 mV, $g_{Ca}=4\text{ mS}/\text{c}{\text{m}}^{2}$, *V*_*ca*_ = 100 mV, $g_{K}=8\text{ mS}/\text{c}{\text{m}}^{2}$, *V*_*K*_ = −70 mV, *V*_1_ = 0 mV, *V*_2_ = 15 mV, *V*_3_ = 10 mV, *V*_4_ = 10 mV, $\bar{\lambda }=0.1\;{\text{s}}^{-1}$, and *I*_*s*_ is the applied scaled input *I*_*s*_ = *K*_*s*_*P*_*d*_ (with *K*_*s*_ = 60 a.u.).

This post-synaptic dendritic activity *P*_*d*_ is determined based on the weighted sum of the post-synaptic local activities (*P*_post_), reflecting the relative contributions from each synapse to the total dendritic activity, so

(10) $$\begin{array}{l} P_{d}=\sum_{i}G_{i}\;P_{post}^{i} \end{array}$$

where *G*_*i*_ = [0, 1]. The cross-correlation terms in Equation (1) are therefore causally related, due to the fact that *P*_post_ is produced by the same spiking model as *P*_pre_, but weighted. If the weight is such that the cross-correlation *P*_pre_*P*_post_ is high, this will *depress* the synapse, unless the post-synaptic cross-correlation term *P*_post_*P*_*d*_, and the local linear *P*_post_ itself can overcome this effect. Similarly, the post-synaptic dendritic activity is causally related to the post-synaptic activities forming the second cross-correlation term. Therefore, the entire dynamic behavior of the synapse is a direct causal function of its input, in relation to its output contribution to the local neuronal activity. The post-synaptic spiking neuron model can then be activated based on the scaled *P*_*d*_. Scaling is necessary due to the fact that the spiking models are based on specific assumptions for their parameter values. Finally, the weight for the synapse is calculated based on Equations (1, 2). Note that all equations are first order differential non-linear equations. These can be simulated using standard numerical integration methods, where the exact methodology used is not critical, taking care to minimize numerical error.

**Figure 1:**
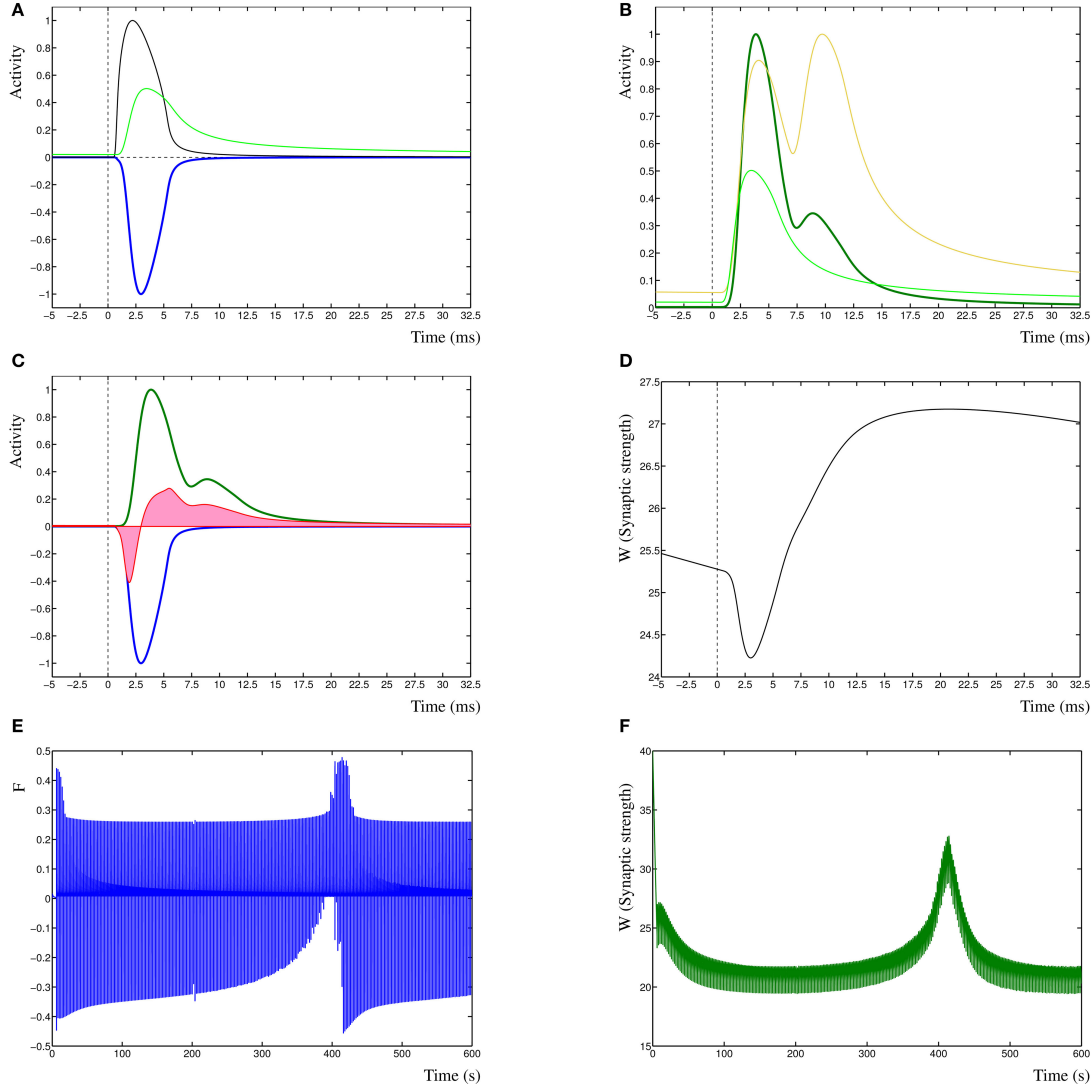
**(A)** Presynaptic activity in black due to a spiking event at time zero, this leads to a local postsynaptic activity (light green). In dark blue is shown the negated cross-correlation of these two activities. **(B)** Postsynaptic activity in light green (same as in **A**) with dendritic activity in gold and cross-correlation of these two in dark green. **(C)** The negative first cross-correlation in dark blue, the positive second cross-correlation in dark green and the learning function *F*(*t*) in red. The pink shading indicates the relative contributions of depression vs. potentiation. **(D)** The synaptic weight *W*(*t*) in time, due to the decay term −*eW*_*i*_(*t*) the synaptic strength is slowly decreasing. **(E)** Function *F*(*t*) over the entire simulation period of 600 s. **(F)** Synaptic weight *W*(*t*) derived from *F*(*t*) in **(E)**.

To clarify the function of the Dynamic Hebbian Learning rule, in Figure 1 are shown the response of the learning rule due to a single spiking event at time zero. In Figure 1A is shown the activity *P*_pre_ (black) and *P*_post_ (green) and the negative cross-correlation of the two (blue, cf. term one in Equation 3). In Figure 1B is shown the same *P*_post_ as in Figure 1A (green) as well as the *P*_*d*_ (gold), resulting in the cross-correlation of these two activities (dark-green, cf. term three in Equation 3). In Figure 1C is shown the two cross-correlation terms from Figures 1A,B (blue and dark green), which results in the learning function *F*(*t*) (red). The synaptic weight *W*_*i*_(*t*) is then shown in Figure 1D, in this case, the input leads to a synaptic strengthening. Even though each individual spiking event has an effect on the model, it does not necessarily by itself cause a change in synaptic strength, unless the synapse is in a critical state where previous events have driven the dynamic system toward the change. A critical state indicates that the system is near a change in dynamic state, such as one stable state to another, or to a periodic oscillation. This is comparable to the recognized critical properties of neural architectures (Abbott and Rohrkemper, 2007). In fact, this property increases the robustness of the system, by not only depending on specific events, but on those events that contribute to a change in state, which makes those events relevant to learning in the synapse.

Finally, in Figure 1 are shown a single sample run of the changes in the learning function *F*(*t*) (Figure 1E), and the synaptic weight *W*_*i*_(*t*) (Figure 1F) over an entire extended simulation period, demonstrating the adaptation of the synaptic weight due to temporal sensitivity to cross-correlated input.

### 2.3. Synaptic configurations

**Figure 2:**
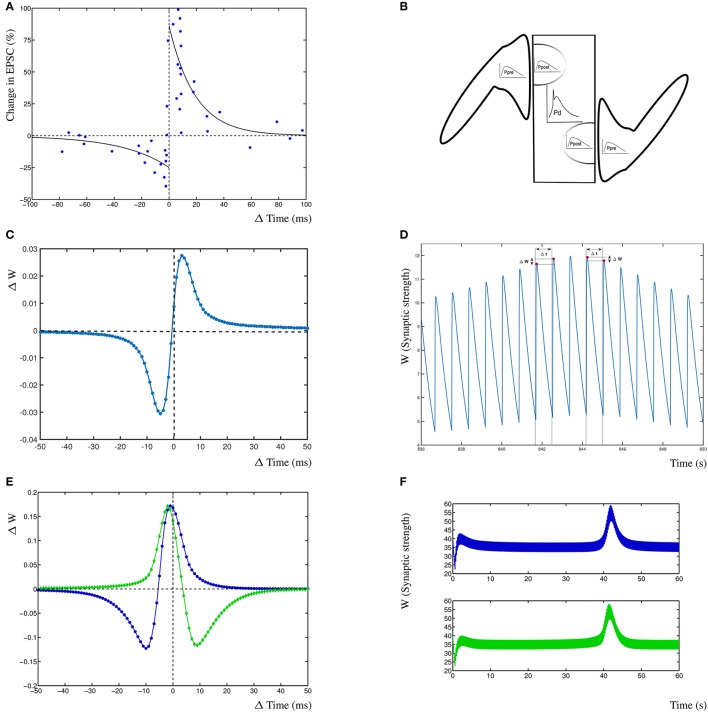
Results from the dynHebb model. **(A)** STDP in hippocampal experiments demonstrating the standard STDP form (revisualized in blue, after Bi and Poo, 1998). The black lines are fitted exponential functions for comparison (redrawn after Bi, 2002). **(B)** Scheme of simulation model where two synapses project onto the same compartment, the presynaptic activity (*P*_pre_) generates local postsynaptic activity (*P*_post_) which contributes to the dendritic activity (*P*_*d*_). **(C)** STDP of 1 Hz stimulated single synapse model. The low change of weight is due to the low activation pattern. **(D)** Synaptic weight of the 1 Hz stimulated model over time, showing four distinct stimulation events, and the subsequent change in weights. **(E)** The dynHebb learning of competing synapses 1 (blue) and 2 (green). **(F)** Synaptic strength of both synapses over one entire simulation period, driven by 5 Hz periodic signal with 1 ms beat. Note that the synapses are stimulated every 200 and 201 ms respectively which results in the thickness of these curves.

The dynHebb rule can be applied to synapses in different spatial configurations. Commonly, two synapses are targeted on the same postsynaptic compartment (illustrated in Figure 2B) and are driven singularly to demonstrate the effect of learning in a single synapse, or by paired periodic pulses with different frequencies. For example, a typical pair of periods are 200 and 201 ms for at least 60 s. This will introduce a beat into the frequencies (a difference between the two frequencies), such that every phase difference between the two periods is contained within the total simulation time. This allows for convenient analysis of the STDP properties. The dynHebb learning rule combines the relative contributions of the synapse and postsynaptic dendrite where the relative difference between the periods determines the time lag. It is more convenient to use a systematic, rather than a stochastic approach; the scheme does not need to have repeated fixed stimulation pairs, as is required in an experimental setup. The determinism of the function (Equation 1) ensures that a single event will cause a very small chance in the synaptic strength. If the learning strength parameters are chosen suitably small, this will not be noticeable, unless the stimulation is repeated conform an experimental approach. A different synaptic configuration is shown in **Figure 7A** of a configuration with two excitatory synapses and one inhibitory synapse. The three synapses are at different distances from the soma, but close enough to each other to influence the local dendritic activity. Synapse A is furthest away, followed by B, and C the nearest to the soma. In subsequent simulations the first synapse from presynaptic neuron A is located on the left branch of the “Y,” the second synapse from presynaptic neuron B is located on the right branch. The third synapse is located on the base of the “Y,” with the location of the soma at the bottom. These synapses will then compete to become the most relevant synapse to cause a postsynaptic activity spike. The synapses will not simply reach the limit of the neuron, due to the relative contribution each makes to the total dendritic, and thus somatic activity. To demonstrate the dynamic nature of the STDP function, we will compare the effects of the different locations of the sole inhibitory synapse. In other words, we make synapse A, B, and C inhibitory in turn, with the other two excitatory.

### 2.4. Determination of relative spike timing

To calculate the resulting STDP curves, a standard experimental approach is used (Verhoog et al., 2013), where the timing of peak of the presynaptic activity *P*_pre_ at sample time *t* is subtracted from the timing of the resulting postsynaptic activity *P*_post_ to provide the relative difference (*P*_post_(*t*)−*P*_pre_(*t*)), and the corresponding amplitude change between the activity events provides the relative change in synaptic strength due to this activity Δ*W*_*i*_(*t*) = *W*_*i*_(*t*) − *W*_*i*_(*t* − 1), where the value of *W*_*i*_ is constant between spiking events. The potential effect of cumulative increases due to a residual memory (state) in the synaptic strength at each increase in the phase difference can easily be mitigated by providing sufficient time between input pulses to allow transients to die out. The dynHebb learning rule is not affected by the current state of the synaptic weight itself, as can be seen from the synaptic rule function $F_{\text{dyn}}^{i}\left(t\right)$. The linear decay −*eW*_*i*_(*t*) in Equation (2) slowly decreases the weight, which can be overcome by occasional activity of the synapse. Alternatively, this term can be set to zero, if desired, without compromising the inherent properties of the rule allowing more persistent activity.

The use of the continuous stimulation protocol with the beat, does not change the properties of the model. Each individual stimulus causes a small change in the synaptic strength, which will persist and become significant if sufficient stimuli occur before this change decays over time. Therefore, the stimulation pattern is not responsible for the learning effect, but the dynHebb rule, and this may be a useful approach for experimentalist as well.

There is therefore no cumulative increase or decrease in the synaptic strength caused by the previous input, unless transient activity causes the cross-correlation terms to be non-zero. This is one of the main features of the dynHebb rule, allowing activity patterns to influence synapses only when they are active. When they are inactive, the presence of dendritic activity does not affect the rule as such, which guarantees the synaptic stability and prevents the occurrence of synaptic degradation associated with high neural activity that may occur with linear and non-linear learning rules.

## 3. Results

To demonstrate the dynHebb learning rule, we used a protocol independent mechanism to determine the relative contribution of each spiking event to the local dynamics of the synapse. Synapses were stimulated with an input pulse driving a modified Morris-Lecar model (Izhikevich, 2004) together with a postsynaptic dendritic pulse at appropriate varying time delays. The resulting change in weight for both synapses mirrors the standard STDP curve for the first synapse and the inverse curve for the second synapse (Figure 2C, explained in detail in the section Synaptic Competition). This can be compared with the reproduced experimental data from Bi and Poo (1998) (Figure 2A). Additionally are shown, the exponential curves that are commonly used to describe STDP learning (continuous lines) as a non-autonomous decay model (Song et al., 2000). These exponential functions are fitted by $\Delta W^{\pm }=A^{\pm }\exp\left(\frac{\Delta t}{\tau^{\pm }}\right)$ with *A*^±^ = 0.86, −0.25 and τ^±^ = 19, –34 (Bi, 2002). In Figures 2C,D are shown the effect of the dynHebb model of the simple experimental protocol by Bi and Poo (1998), where the synapse is activated by a 1 Hz frequency with a 1 ms beat. The STDP diagram in Figure 2C shows the classic STDP curve, without any further adjustment, although the change in weight is very small, due to the large gaps between each subsequent activation. In Figure 2D is shown the synaptic weight change due to the four stimulations causing small changes in the weight. To demonstrate the effect of more closely related activation patterns, two nearby synapses were simulated at 5 Hz with a period difference of 1 ms. The dynHebb learning rule response dynamically to the change in configuration, as is shown by synapse 1 (Figure 2E blue line), which contains data points that cause depression, as well as potentiation. The synaptic strengths of both synapses do not change when the two synapses are out of phase and the cross-correlation terms are zero. The synapses change their relative strengths when both input and additional dendritic activity are present due to non-zero cross-correlations. In Figure 2F at about 40s is shown the pre- and postsynaptic activity to become in phase, resulting in the increase of synaptic strength.

### 3.1. Synaptic competition

Synaptic competition is a cardinal feature of STDP learning that is associated with synaptic pruning. This has been shown to occur due to repetitive LTD induction in rat hippocampal neurons (Shinoda et al., 2010). It has also been demonstrated that, for short time windows, spines may compete for L-LTP expression (Govindarajan et al., 2011) and competition was found for different signaling molecules (Gerkin et al., 2007). It is therefore expected that any functional learning rule will show input specific responses for plasticity rules (Feldman, 2000) between nearby synapses on the basis of postsynaptic activity interactions.

To demonstrate competition in our model, two synapses are simulated and driven with two periodic signals of 5 Hz (200 ms period) and 4.975 Hz (201 ms period), resulting in a beat frequency of 0.025 Hz and a total stimulation period of 40 s. No further dendritic activity exists apart from the activity generated by either of the synapses 1 and 2. Competition can be found in this situation as interacting synaptic strengths over an entire simulation period (Figure 2F). The second synapse increases in strength before the first synapse due to the difference in phase of the beat.

The subsequent change in phase difference causes the first synapse to increase in strength and the second synapse to weaken again (at 42 s). Finally, the first synapse will weaken and both synapses return to their equilibrium state. From this simulation, the STDP curve can be determined by calculating the time differences for individual inputs with the postsynaptic response. The resulting STDP curves of both synapses show that the first synapse (Figure 2C blue) has the traditional Hebb type STDP learning curve and the second synapse 2 (Figure 2C green) has an anti-Hebbian shape due to the fact that the first synapse has a slightly higher frequency and over time is more often active. This shows that competing synapses may have different STDP learning curves simply due to the difference in timing induced by the presence of postsynaptic activity of other synapses.

### 3.2. Frequency dependence

**Figure 3:**
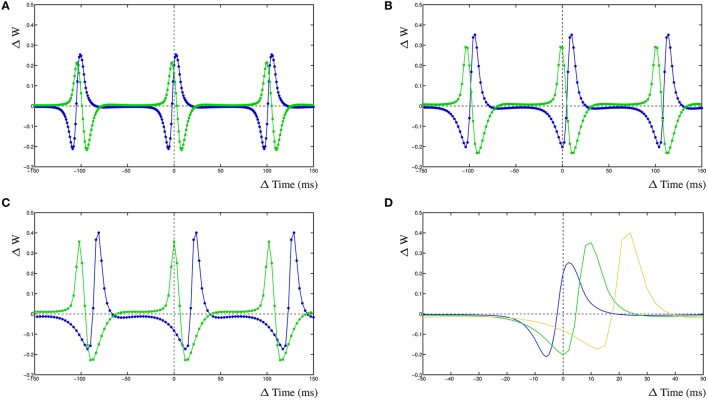
Frequency dependence of two synapses, STDP curve plots with varying input frequencies for synapse 1 (blue) and synapse 2 (green). **(A)** Input frequencies of 10 and ~9.9 Hz (1 ms difference). **(B)** Input frequencies of 10 and ~9.8 Hz (2 ms difference). **(C)** Input frequencies of 10 and ~9.7 Hz (3 ms difference). **(D)** STDP curves of the first synapse receiving 10 Hz input drawn from **(A–C)**, changes are solely due to the postsynaptic dynamics induced by the second synapse. From left to right, second synapse input is ~9.9, ~9.8, and ~9.7 Hz.

The dynamic interactions that emerge from the dynHebb learning rule, contribute to more complex behavior of the synapse. Because firing rate and timing as well as correlation have been shown to contribute to cortical plasticity (Sjöström et al., 2001), different input frequencies are expected to modify the resulting STDP curve. We demonstrate this in the dynHebb model by driving the two synapses with different input frequencies in the absence of further postsynaptic activity than generated by the synapses themselves. Given an input frequency of 10 and ~9.9 Hz respectively (with 1 ms difference), the resulting STDP (Figure 3A) is already different from the previously described results, where the input frequency was 5 Hz (Figure 2C), due to a significant shift of the STDP curve. Subsequently, driving the same two synapses with a frequency of 10 and ~9.8 Hz (2 ms difference), causes a larger shift of the STDP curve (Figure 3B). Finally, driven by 10 and ~9.7 Hz (3 ms difference), the two STDP curves have shifted further (Figure 3C), which demonstrates the effect of relative timing. This is summarized in Figure 3D where the STDP curves of the first synapse alone are plotted together. Notice that the STDP curve of the first synapse has changed due to the difference with the input period of the second synapse. This nicely illustrates the importance of the postsynaptic dynamics for the synapse and how a synapse adapts its own dynamics by relating its received input with the local postsynaptic dynamics.

### 3.3. Action potentials

Another important contribution to the emerging STDP curve is the presence of postsynaptic action potentials. For this paper, no particular distinction is made between the different sources or directions of the action potential, merely the effect such an event has on the local dendritic activity and, subsequently, on the local synapse. The insensitivity of the synapse to the source of the action potential illustrates the relative independence of the synapse. It only knows about local observable events, but is influenced by other events further away which affect the postsynaptic activity to which the synapse adjusts itself. Using the same configuration as used previously, two synapses are driven with slightly different frequencies but also an additional independent postsynaptic activity is added with a separate related frequency. To demonstrate the relative contribution of an action potential to the shape of the STDP curve, the simulated action potential had a frequency that was slightly higher or lower than the input frequency to either of the two synapses.

**Figure 4:**
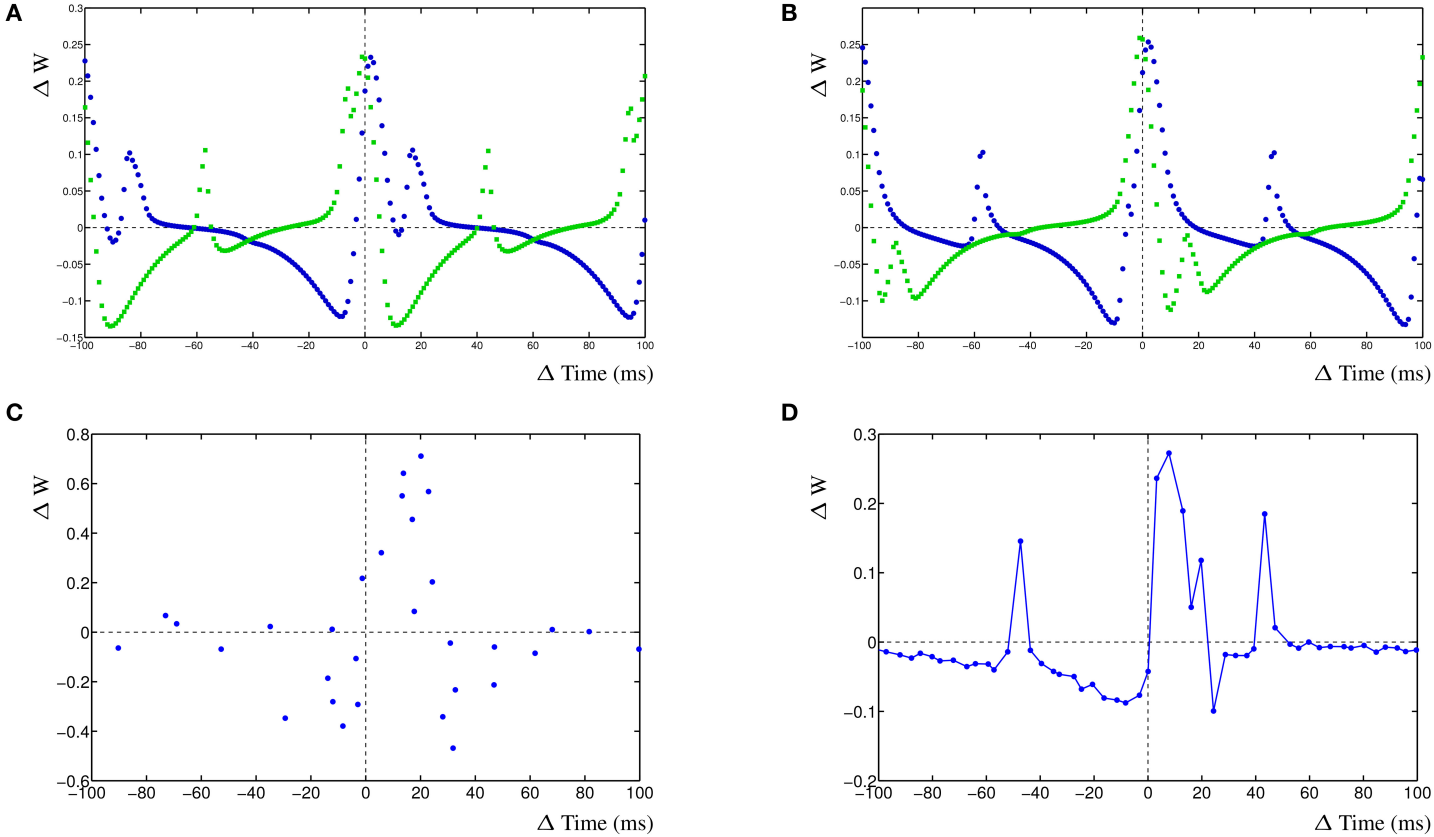
Effect of postsynaptic action potential on synapses. **(A)** STDP curve with postsynaptic action potential with frequency ~10.1 Hz (99 ms) and two competing synapses, synapse 1 has an input frequency of 10 Hz (100 ms) and synapse 2 has an input frequency of ~9.9 Hz (101 ms). **(B)** STDP curve with postsynaptic action potential with frequency ~9.8 Hz (102 ms) and two competing synapses, synapse 1 has an input frequency of 10 Hz (100 ms) and synapse 2 has an input frequency of ~9.9 Hz (101 ms). **(C)** STDP plot, revisualized after Wittenberg and Wang (2006), showing a shifted LTP window and two LTD windows, stimulation frequency was 5 Hz. **(D)** Single compartment simulation of two synapses, one stimulated at 5Hz, one at 4.9 Hz and a BAP at 4.78 Hz. It results in a shifted LTP window and two LTD windows in the STDP curve. Also shown are two harmonic peaks at +43.35 and −47.3 ms that are not in the experimental data.

For the first case, synapse 1 was driven with a frequency of 10 Hz, synapse 2 with a frequency of ~9.9 Hz (1 ms difference) and the action potential was driven by a frequency of ~10.1 Hz (99 ms). When the simulated action potential has a slightly higher frequency than the two synapses (Figure 4A), there are several additional LTP windows for either of the two STDP curves corresponding to the two synapses. Synapse 1 has an additional LTP maximum at ≈+18 ms and synapse 2 has several additional LTP maxima, at ≈−3 ms, ≈−58 ms and ≈+44 ms. When the action potential frequency is equal to that from synapse one, the shape of the STDP curve of synapse 1 has not changed, compared to the simple competition between two synapses without an additional action potential. The only difference is that the amplitude of the STDP curve has increased somewhat (not shown). The STDP curve for synapse 2 has an additional LTP peak at ≈−4 ms. This gives the appearance that the LTP window of the STDP curve for synapse 2 is wider than when the action potential has a higher frequency. Interestingly, when the frequency of the action potential is further decreased and becomes the same as that of synapse 2, the resulting STDP curve is not much different from the STDP curve without an action potential, apart that the amplitude of the STDP curve is higher (not shown). This is due to the fact that both synapses provide postsynaptic activity and the action potential coincides with these two activities which does not contribute much to the learning needed of either synapse. Lastly, when the frequency of the action potential is lowered such that it is smaller than either of the two synapses at ~9.8 Hz (Figure 4B), the STDP curve for synapse 1 has two additional LTP maxima, at ≈−58 and ≈+48 ms. Synapse 2 has now two additional LTD minima at ≈−82 and ≈+23 ms.

### 3.4. Inhibition

**Figure 5:**
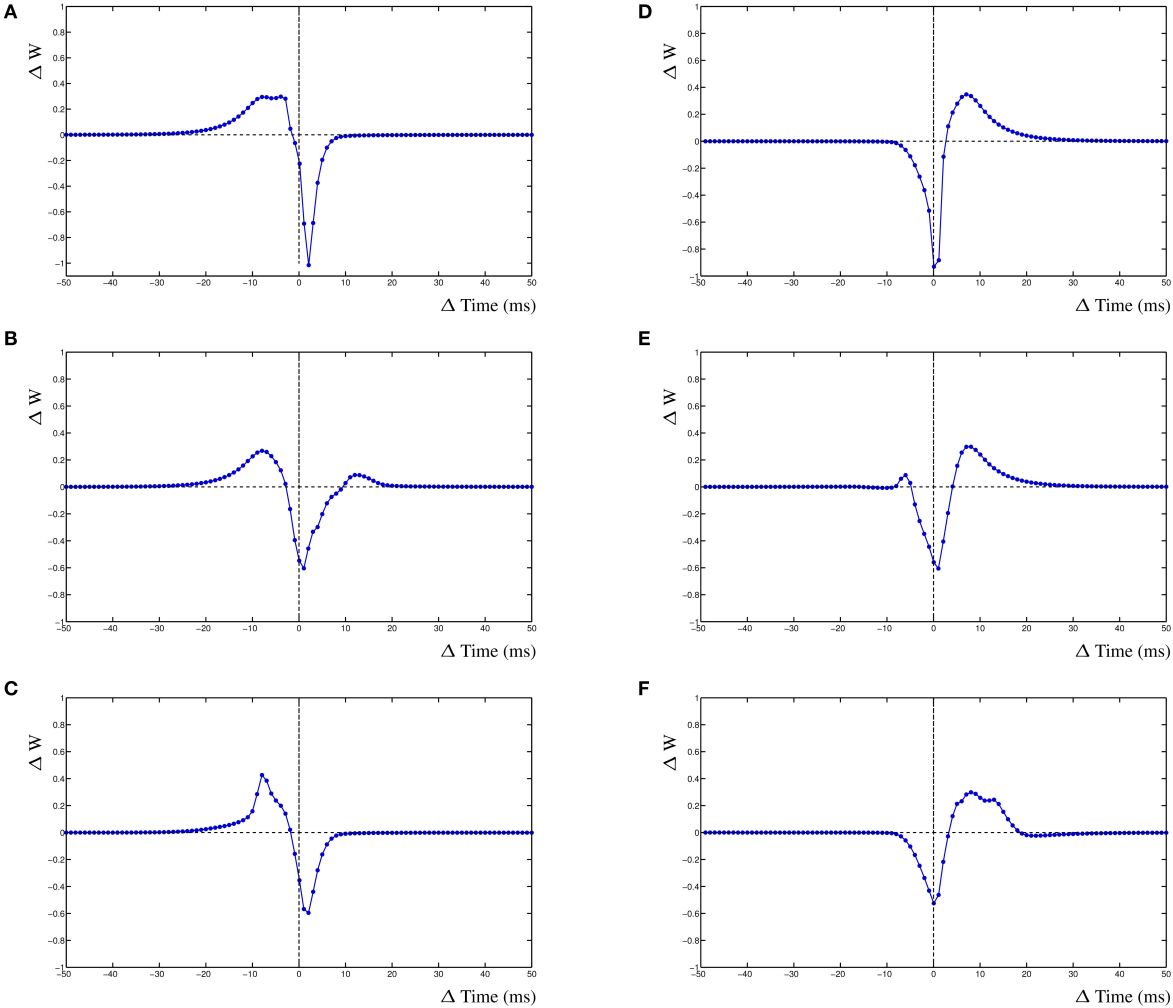
Inhibition and the STDP curve. A single excitatory synapse and a single inhibitory synapse interact by modifying the postsynaptic activity. A postsynaptic action potential causes further adaptation. Shown is the STDP curve from the excitatory synapse. Left column **(A–C)** STDP curve of excitatory synapse driven by 5 Hz (200 ms) input with ~4.98 Hz (201 ms) inhibitory input. In **(A,D)** no further postsynaptic activity. In **(B,E)** an additional postsynaptic action potential at ~5.025 Hz (199 ms); in **(C,F)** the action potential has a frequency of ~4.95 Hz (202 ms).

The dynHebb learning rule employs cross-correlation to combine the input activity with the local postsynaptic dynamics. Synaptic inhibition affects the amount of postsynaptic activity and therefore will influence the STDP curve depending on the timing of the inhibitory input. Additionally, the occurrence of a simultaneous action potential can modify the STDP curve further. To demonstrate these interacting activities, a different configuration has been used. In a single compartment, one excitatory synapse is driven using a fixed frequency. A nearby inhibitory synapse is driven using a different frequency. An optional postsynaptic action potential can also be included, and subsequently, the resulting STDP curves are determined (Figure 5). The excitatory synapse is driven using a frequency of 5 Hz (200 ms) and the inhibitory synapse with a frequency of ~4.98 Hz (201 ms) (Figure 5A–C left column). Alternatively, the excitatory synapse can be driven by a frequency of ~4.98 Hz (201 ms) and the inhibitory synapse by 5 Hz (200 ms) (Figure 5D–F right column).

We first regard the STDP curves of the excitatory synapse without any further postsynaptic activity, such as an action potential, but with inhibition (Figures 5A,D). The presence of the inhibitory activity causes the curve to be shifted, widened and inverted. The normally Hebbian STDP curve has become an anti-Hebbian curve and the LTP window is larger. Similarly, the presence of inhibition has caused the anti-Hebbian curve to become inverted as well, also the LTP window has widened and the curve has shifted (Figure 5D). If the postsynaptic action potential is included when the excitatory synapse has a higher frequency than the inhibitory synapse, the shape of the STDP curve changes yet again. When the action potential occurs at a frequency of ~5.025 Hz (199 ms), a second LTP window appears at ≈+12 ms (Figure 5B). If the frequency of the action potential is changed to ~4.95 Hz (202 ms), the single LTP maximum has significantly increased but is less wide (Figure 5C). Adding a postsynaptic action potential when the inhibitory synapse has a higher frequency than the excitatory synapse, a similar but mirrored STDP curve is found. A second LTP window may also appear at ≈−6 ms (Figure 5E). This second LTP window may disappear again with the lower frequency of the action potential in that case (Figure 5F).

### 3.5. Changing shape of postsynaptic activity during development

During development, due to the changing properties of channel dynamics such as NMDA-channels, the shape of the postsynaptic EPSP changes (Quinlan et al., 1999; Wittenberg and Wang, 2006; Meredith et al., 2007; Sjöström et al., 2008). In particular, the width of the EPSP becomes smaller, probably due to a change in decay times. We will use the competing synapse configuration to study the effect of different widths of the EPSP by changing the decay rate such that in the young neuron simulation, the EPSP is higher than in the mature neuron simulation. Subsequently, we will add input noise onto the synaptic inputs and compare the results.

**Figure 6:**
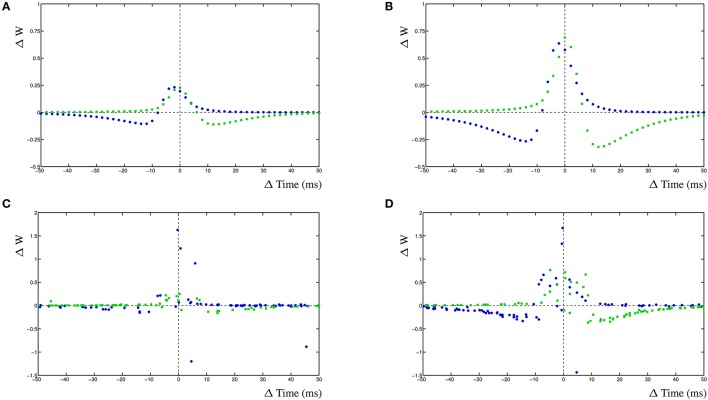
Change in EPSP shape during development of two competing synapses. **(A)** STDP curves for relative shorter EPSP shape, comparable to adult, without noise. **(B)** STDP curves with relative wider EPSP shape, comparable to young neuron, without noise. **(C)** STDP curves for relative shorter EPSP shape with added 20% input noise. **(D)** STDP curves for relative longer EPSP shape with added 20% input noise.

For the simulation of a mature neuron, the STDP window has fairly narrow windows (Figure 6A). The comparable STDP curve for the young neuron simulation, with halved decay rates, has much higher STDP windows (Figure 6B). It is immediately obvious that wider EPSPs will lead to larger cross-correlation windows and therefore to a higher STDP window. The increase in amplitude is caused by the overall higher activity in the postsynaptic compartment. We subsequently add a 20% input noise onto the adult and young neuron simulations. This results in a significant number of event that are mis-timed and the STDP window becomes less well defined as the cross-correlation is less relevant for the adult neuron (Figure 6C). Lastly, we also add a 20% input noise onto the young neuron simulation. In this case, the STDP windows are still fairly effective and can successfully be used to train the neuron on noisy input which results in a stable network (Figure 6D).

### 3.6. Competing synapses at the local dendrite

**Figure 7:**
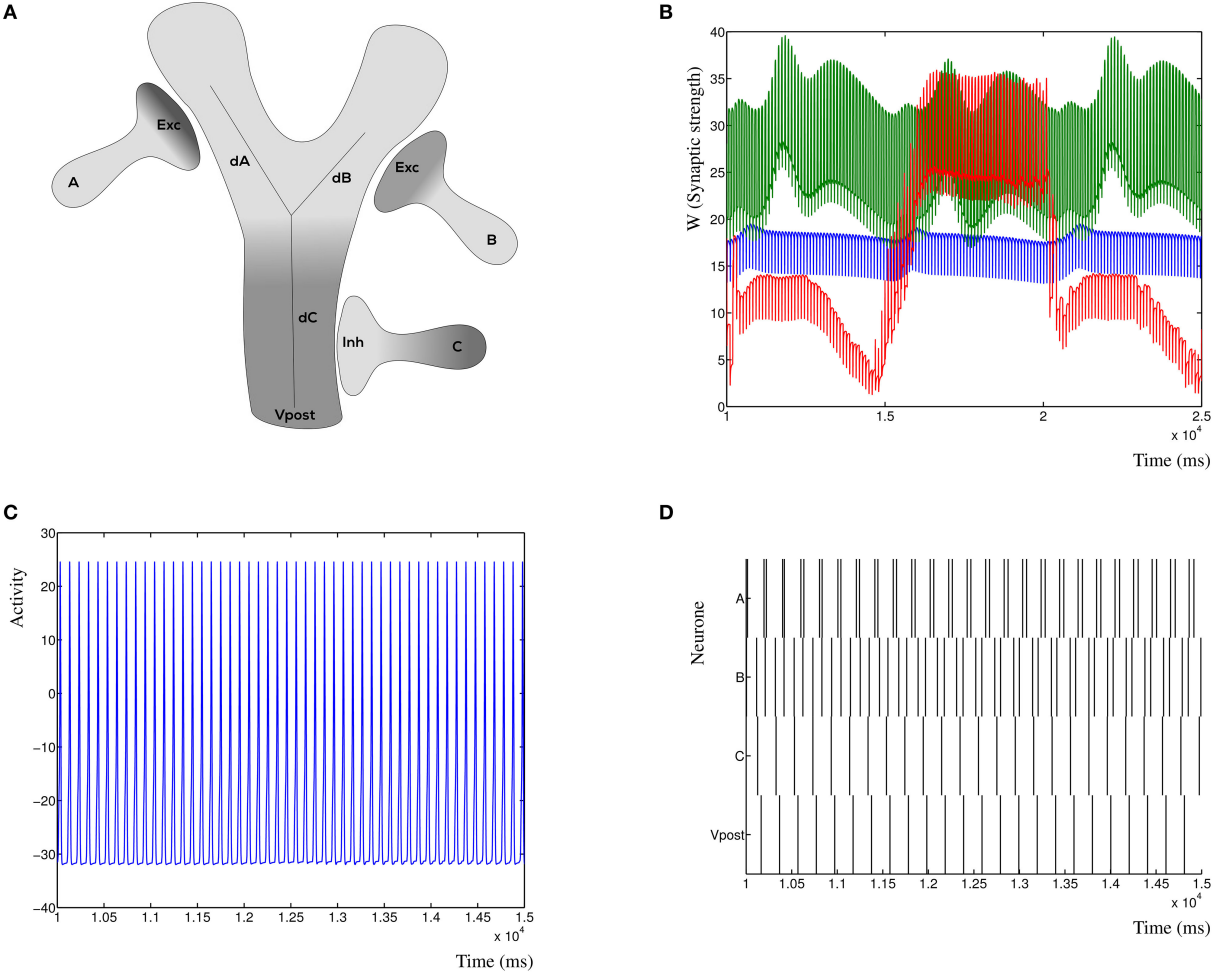
Synaptic competition at the dendrite. **(A)** Three simulated synapses compete to activate the postsynaptic neuron. Each of the synapses can be chosen to be excitatory or inhibitory, here is shown the configuration with the distal synapses as excitatory. The distances *dA, dB*, and *dC* indicate the relative attenuation of the activation for each contributing synapse. **(B)** Synaptic strength variation due to three competing excitatory synapses a small distance from each other. **(C)** Detail of the postsynaptic response due to three excitatory inputs. The response is dynamically stable. **(D)** Raster plot of the three presynaptic input patterns (A, B, and C) where all are excitatory, and the resulting postsynaptic response. Notice that the resulting post-synaptic pattern is dynamically stable and not merely the sum of the inputs.

To demonstrate the inherent stability of the dynHebb learning rule, we simulate three synapses (Figure 7A), which are all excitatory. Each synapse has its own frequency, and the resulting postsynaptic activity is therefore a combination of the cross-correlated inputs. In Figure 7B is shown the resulting dynamic synaptic strength. The synapse furthest from the soma shows the least variability (green) due to the relative independence of this particular synapse. The synapse nearest, on the other hand, may vary a lot when the other two have a relatively strong (or weak) effect on its own cross-correlation (red). Changing the input frequencies will not cause the synapses to explode or disappear over time. The resulting postsynaptic activity is shown in Figure 7C, which shows a simple regular output pattern. This can be readily compared to the presynaptic input activities in Figure 7D, where the raster plot shows the correlation patterns of the three input synapses and the postsynaptic activity.

**Figure 8:**
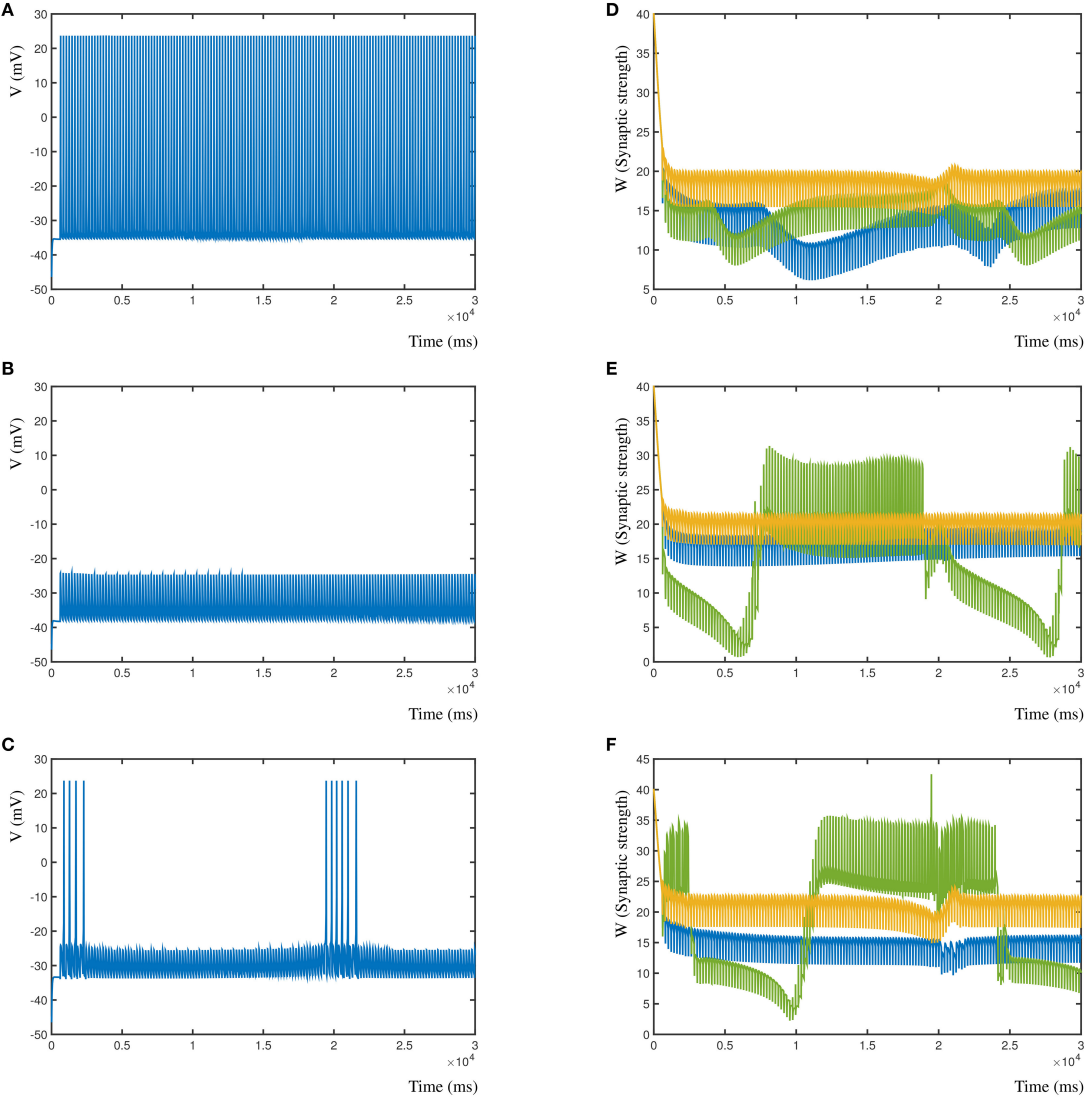
Effect of different relative locations of the inhibitory synapse in relation to two excitatory synapses. **(A)** The post-synaptic voltage of the Morris-Lecar neuron when synapse A is inhibitory. **(B)** Post-synaptic voltage when synapse B is inhibitory. **(C)** Post-synaptic voltage when synapse C is inhibitory. **(D)** Synaptic strengths over time when synapse A is inhibitory. **(E,F)** Same for synapses B and C.

Conversely, in Figures 8A–C (left column), is shown the resulting post-synaptic voltage of the Morris-Lecar neuron when the sole inhibitory synapse is A (Figure 8A), B (Figure 8A), and C (Figure 8C). As expected, based on the relative distance, inhibitory synapse A is too far away to affect the excitatory activity patterns of B and C. Similarly, inhibitory synapse B suppresses the entire post-synaptic neuron, where A is not strong enough to compensate for B. Lastly, the proximal inhibitory synapse C suppresses the activity, except when distal excitatory synapses A and B correlate to overcome the inhibition. In all cases the synapses where activated with the same frequency, independent of their inhibitory or excitatory nature (A = 5 Hz, B = 5.025 Hz, and C = 4.9 Hz). In Figure 8D–F (right column) are shown the corresponding changes in synaptic weight due to the competition in the three synapses. Here, synapse A is shown blue, B is green and C is gold.

## 4. Discussion

Donald Olding Hebb's thesis on synaptic plasticity can be explicitly expressed as the change in synaptic efficacy in proportion to the degree of correlation between pre- and postsynaptic activity (Hebb, 1949). The Hebbian learning rule has been demonstrated in computer simulations to be reliable and effective in determining synaptic strengths and appears to have a solid foundation in experimental biology (Bi and Poo, 1998; Meredith et al., 2007). However, beyond curve fit models of the STDP rule, commonly expressed as two exponential decay curves (Song et al., 2000), it has not been demonstrated that the causal correlation property of synaptic plasticity is valid, and is effective as a learning mechanism as may have been assumed. Indeed, the focus on spiking models and synaptic STDP models based on curve fit approximations has detracted from this cardinal aspect of learning. A lot of effort has been put into ephemeral problems associated with the modeling rather than the STDP mechanism itself and its relevance to learning in biology (Morrison et al., 2008). In particular, extensive spike sorting algorithms have been defined to resolve the issue of timing relevance of pre-after-post vs. post-after-pre spiking patterns which are, in effect, an STDP version of supervised learning. Biochemical synaptic processes are autonomous and dynamically complex, from which time based activity emerges (olde Scheper, 2008). The dynHebb model eliminates explicit time which makes it possible to determine the relative temporal contributions of synapses to network learning in a consistent and independent manner. The independence of the model, with regards to explicit time windows, allows greater flexibility and adaptability for simulating different synaptic input types, if wishing to simulate their STDP curve ranges. The lack of an explicit time window does not force the synapse to behave to discrete pairing windows but rather works on the causal correlation of synaptic activity in a dynamic manner. Therefore, the model clearly demonstrates that time dependent processes do not require an intrinsic clock or event detection but are sensitive to temporal codes simply due to deterministic autonomous dynamic behavior.

The development of a phenomenological learning rule appears to be somewhat contrary to current interpretations of synaptic plasticity modeling. In particular, various models have been published to attempt to explain STDP in terms of biochemical processes (e.g., Sjöström et al., 2001; Graupner and Brunel, 2012). In this paper, we do not claim to explain the biophysics of STDP, but attempt to provide a mechanistic approach, which is not simplistic, but powerful enough to address the main criticisms that STDP modeling attracts (Shouval et al., 2010). Specifically, the dynHebb model allows a minimal model with sufficient, and necessary, complexity to allow comprehension of the key concepts, without specific biochemical interpretations that form the basis of many arguments about interpretation.

### 4.1. The dynamic hebbian cross-correlation rule

The dynamic Hebbian cross-correlation learning rule is a conceptual activity measure that is not bound to any biophysical feature beyond the implied causality imposed by the synaptic chemical organization. Even though different interpretations have been given to specific roles and functions of biophysical elements, such as calcium and neurotransmitter receptors (Clopath et al., 2010; Graupner and Brunel, 2012), the relative importance of those specific elements to the entire complex system is still contentious. We argue, therefore, that the synaptic learning is based on dynamic nonlinear interactions between biophysical systems and that attributing specific STDP properties to chosen biophysical elements is an over-interpretation of the available data. To encompass the entire dynamics, it is more suitable to a phenomenological interpretation that allows the high level STDP dynamics to be described. Similar approaches have been previously employed extensively in neuromodeling (Koch, 1999), indeed the classical STDP model curve is a phenomenological curve fitted to the data (Song et al., 2000).

The interaction between the depression due to input and potentiation due to postsynaptic activity dynamically determines the amplitude and width of the STDP windows. For a single synapse, with no further postsynaptic activities than the dendritic pulse it generated, the emerging shape is the well known Hebbian STDP learning window. As we will show, variations in timing, amplitude and width of the STDP curve are solely due to the relative difference in cross-correlation timing pre-synaptically vs. post-synaptically. Electrical properties of individual synapses and morphological properties of dendrites may result in different variations of the STDP window. If other synapses are near enough to affect the local dendritic activity near the synapse under investigation, they can have a significant effect on the STDP curve of that synapse. Additionally, other waves of activity, such as Back-propagating Action Potentials (BAP) and dendritic activities, will also affect the postsynaptic activity of the synapse. This can be determined by examining well known properties of STDP as described below.

### 4.2. Synaptic competition

The apparent change in STDP learning rule induced by the difference in external input timings has also been reported to occur experimentally in CA1 pyramidal neurons where the external input timing at local synapse determines the sign of STDP learning (Kwag and Paulsen, 2009). The competition of the synapses illustrates an important feature of the dynHebb model and its implications for STDP learning. The crux is the interaction between the local dynamics of the synapse itself with the global or wider local dynamics of nearby synapses and the neuronal local state as expressed by an action potential in the dendrite.

It has been suggested that different STDP windows may be specific for different cell types, such as in the dorsal cochlear nucleus (Tzounopoulos et al., 2004) where Hebbian and anti-Hebbian type STDP windows have been found for different cell types. In such cases, postsynaptic activity may induce both Hebbian and anti-Hebbian STDP windows depending on the local postsynaptic dynamics due to interactions of various sources of activity. Multi-synaptic activation can readily skew the expected results depending on the local flow of currents. This can be seen in the example of the competing synapses where synapse 2 (Figure 2C green line) has an anti-Hebbian STDP window due to the competing activity of synapse 1. It was also shown experimentally that the location of synapses within the dendritic tree is relevant to the form of the STDP learning rule (Froemke et al., 2010). This is predicted by the dynHebb learning rule, as in more distal areas of the dendritic tree, more complex postsynaptic interactions are possible when BAPs contribute significantly less to the STDP learning rule. The proximal synapse is therefore still largely influenced by the relatively strong contribution of the BAP to the postsynaptic activity. Experimental results that describe the loss of temporal contrast due to dopaminergic modulation of STDP (Zhang et al., 2009) can thus be explained by changes in the responsiveness of the competing cross-correlation based depression and potentiation. Furthermore, it has been shown that integration of synaptic input is governed by local recruitment of NMDA receptor channels (Larkum et al., 2009), demonstrating the importance of local interaction at the postsynaptic site for learning.

### 4.3. Frequency dependence

The results on frequency dependence show that the dynHebb correlation learning rule also applies to harmonics. For the input frequencies around 10 Hz, the shape of the STDP curve at ±100 ms (e.g., Figure 3A) is comparable to the curve around the origin. It is possible for the harmonic curves to have somewhat different shapes due to differences in timing of the harmonic input with the postsynaptic activity. This has already been shown in both simulations as well as experimental data (Meredith et al., 2007; Clopath et al., 2010, supplement) and it has been suggested that resonance may have a functional role in neuronal communication (Izhikevich et al., 2003). The oscillatory control of STDP in mushroom bodies in the locust may well be a fundamental facet of neuronal network function in many species (Kwag and Paulsen, 2009). Additionally, the importance of harmonics in STDP is relevant to the reported variability of STDP learning rules whose synaptic location on the dendrite modifies the STDP curve (Letzkus et al., 2006; Froemke et al., 2010).

The existence of heterosynaptic plasticity has been shown in various experiments (Morrison et al., 2007; Abraham, 2008). In particular, the change in one synapse appears to prevent a corresponding change in other synapses (Abraham, 2008) and depends on conditioning frequencies (Wang and Wagner, 1999), which cause a significant shift of the STDP window in heterosynaptic plasticity. This is comparable to the apparent shift in the STDP window in the model of two competing synapses where the activity of one synapse prevents the second synapse to become potentiated at the same timings and the difference in input frequency causes a temporal shift of the STDP window (Verhoog et al., 2013).

Additionally, the dendritic organization of cortical input *in vivo* has been shown to be organized locally which allows the integration of spatially distributed input (Jia et al., 2010). The dynHebb model demonstrates that local interactions of competing synapses can readily result in this type of integration by combining temporal information from spatially separate synapses.

### 4.4. Action potentials

The frequency and relative timing dependent nature of the contribution of the action potential to STDP has been shown to occur widely (Sjöström et al., 2008). A typical example is the malleability of the CA1-CA3 synapse, as has been shown by Wittenberg and Wang (2006) (Figure 4C). They demonstrated that with increased timing differences of the BAP other than 1 ms, varying amounts of plasticity can be shown, including multiple LTD windows. Without the quantitative data, we can only compare qualitatively these experimental results, to a simulation of the single compartment model with two synapses and an action potential (Figure 4D), which is the provided interpretation of the experiment (Wittenberg and Wang, 2006). They result in a STDP curve with a single time shifted LTP window with two LTD windows and two additional resonance peaks. It was produced with one synapse stimulated at 5 Hz, one synapse stimulated at 4.9 HZ (204 ms) and an action potential at 4.78 Hz (209.1 ms). It may be concluded that the interaction of the different sources of activity can readily result in different STDP curves where local synaptic properties determine the exact shape of the curve in combination with the timing differences of those sources at the synapse.

These results illustrate the need for synapses to receive global input from the neuron itself to scale the synaptic input and to receive input from other synapses but not to be overwhelmed by the total postsynaptic activity. Interaction between synapses via the postsynaptic activity generated by other synapses and action potentials permits a wide range of possible behaviors of the STDP learning curve. Using local synaptic rules which integrate the information from other sources ensures stability of both the neuron and the network (Marder and Goaillard, 2006; Sjöström et al., 2008).

### 4.5. Inhibition

The clear effect of the interaction of the synaptic inhibition with the STDP learning rule is that the associated excitatory input in the synapse should be weakened, rather than strengthened, whenever the inhibitory input occurs after the excitatory input. Additionally, the synapse should be strengthened whenever the inhibitory input precedes the excitatory input. Synaptic inhibition, therefore, not only controls the activity level of the neuron, but also directs the type of learning of nearby excitatory synapses in the target neuron. Experimental results of STDP in the striatum demonstrate the existence of a switch from anti-Hebbian to Hebbian type STDP learning curves in the presence of GABA-ergic blockers (Fino et al., 2005, 2009, 2010; Pawlak and Kerr, 2008). This finding has been suggested to be a more generic property of neuronal networks than previously has been assumed and may readily explain the function of inhibition in synaptic integration and spike timing found in CA1 (Lamsa et al., 2010). Furthermore, synaptic pruning due to repetitive LTD induction found in rat hippocampal neurons (Shinoda et al., 2010) could well be explained by this dynamic role of inhibition on already existing synaptic connectivity.

### 4.6. Activity during development

We suggest that the functional role of the changing EPSPs is related to the relative unreliability of input in the young neuron. The young network has lower discerning resolution but has better reliability in terms of the correct response due to spike timing. The younger neuron can still be trained even with a high level of input noise. In the mature neuron, the more narrow EPSPs result in STDP learning with higher resolution because of the increased reliability of the input due to the training of the entire network. This mechanism could be seen as a reliable biological mechanism for annealing the network into stable and functional networks with high resolution. A similar phenomenon has been observed in dissociated cortex cultures, where wide bursting profiles are predominant in the early stages of growth, and after 3–4 weeks show a short onset, resulting in increased timing precision of the synchronization between neurons (van Pelt et al., 2004).

### 4.7. Local dendritic competition

The dynHebb synaptic model can be employed to investigate interactions between locally competing synapses at the dendrite. This type of localized competition is most important for local computation at the dendritic level (olde Scheper, 2008). Furthermore, this concept is essential to maintain stability of the network, preventing synapses from “exploding” due to large increases in synaptic weight, a common issue in modeling using the classic STDP function. Using a simulation of three interacting synapses we show the functional dynamic response over time of competing synapses with and without inhibition at different distances from each other. The resulting postsynaptic activity is not the sum of all inputs, as the cross-correlation of each synapse interacts with the other synapses at the local level. If all synapses are excitatory, this could readily result in inherently unstable dynamic states, when each of the synapses increases the synaptic strength of the other synapses. In the dynHebb model, this is not possible, because synapses without input do not change their state, and they are limited in the sense that for even higher synaptic weight, the local state of the synapse needs to match the local dendritic activity, which has naturally a maximum. If we also consider the possibility of adding inhibitory synapses to this concept, the number of possible interactions increase even further. When an inhibitory synapse reduces the local dendritic activity, the excitatory synapses will gain greater sensitivity to changes. Extending the number of synapses, the distances between the synapses and the type of synaptic activity will make this system show more complex and interesting behaviors. In particular, the ability to match cross-correlation patterns for different inputs and local activities of different parts of the dendritic tree. The dynHebb model ensures that the synapses remain functional, are attuned to their input, and will not degrade over time, unless negative input correlation occurs.

### 4.8. Model comparison

Although the aim of this paper is not to perform an extensive comparison of the various models of STDP, but rather to emphasize the importance of dynamic models, it would be appropriate to mention some cardinal differences between the dynHebb model and selected popular models. Firstly, models like Shouval et al. (2002) and Graupner and Brunel (2012) emphasize specific biophysical properties to the manifestation of STDP, such as NMDA receptors and intracellular Calcium signaling. Neither paper suggests that these are the sole mechanisms involved in a complex biochemical process, yet by deliberately choosing a single aspect, the impression is given that the other biophysical interactions are of secondary importance. By creating a model that focusses on the dynamic behavior, the dynHebb model avoids this issue, but still allows expansion of the model with each of those biophysical aspects in subsequent work. Secondly, the stability of each of these two models is not ensured when synapses interact, or when there is a large amount of current injected, e.g., due to coinciding activities of nearby synapses. Both models only regard single model synapses, with specific parameter choices to ensure stability. The dynHebb model is inherently robust, self-limiting, and allows complex synaptic interactions. Lastly, both models are based on non-autonomous equations (i.e., dependent on explicit time), which requires spike selecting and fixed responses of the synapse based on the chosen time parameter constants. The dynHebb model causes the emergence of STDP from the timings of the activity functions, where different activity functions cause different time responses.

### 4.9. Experimental implications

Specific STDP learning rules are often experimentally determined by experimental protocols (Testa-Silva et al., 2010). These rules would otherwise not be readily visible in the resulting experimentally determined synaptic behavior. This is indeed a sensible requirement when dealing with a noisy, inexact measurement of a relatively small change in the excitatory postsynaptic potential. However, the concept relies on several premises for which there is only scant evidence. Firstly, the extracellular or presynaptic neural stimulation activates an unknown number of synapses whose combined postsynaptic response is assumed to be negligible at the level of the synapse under investigation. As can be seen from our model results, this is certainly not the case, nor is this desirable for each individual synapse. Secondly, the exact timings in experiments are still tentative given the relatively large margin of error needed to ensure proper response timings, that are not obscured by the experimental protocol itself. Lastly, results are obtained from many different synapses, often in different but identical type of neurons with no consideration for the huge variability that must exist for the local dynamics due to different morphologies and other local properties of the neuron. It is to the skill and persistence of the experimenter that we must attribute that results are found at all, despite the large number of obstacles that need to be overcome.

Obviously, there is no ready made solution that can easily simplify the process, however by assuming a methodology that is more in line with the natural process of synaptic transmission, rather than adhering to a steady-state approach, the likelihood of determining significant results can be greatly increased. The deterministic nature of the dynHebb rule allows the emergence of the small relative contribution of individual learning events without the stochastic background noise that can obscure the event in probability based learning rules. In our simulations, we employ a continuous stimulation protocol that does not require instantaneous updates and highlights the dynamic nature of STDP learning. We can replicate a standard experimental protocol by activating a specific model synapse directly and generate a postsynaptic action potential at the soma. Or, we can activate nearby model synapses to demonstrate the dynamic interaction at the synaptic level of the postsynaptic activity induced by different synapses. This will allow further development of the STDP model using experimental results to guide the specific interpretation of the Hebbian learning.

## 5. Conclusion

The Dynamic Hebbian Learning model demonstrates that only local autonomous non-linear interactions govern the STDP learning rules. The dynamic interactions at the postsynaptic site modify the default Hebbian STDP curve into many possible experimentally observed forms. More detailed modeling of the constraints that determine the shape of the learning curve, such as local dendritic morphology, networks of neurons, current flow within the dendrite and second messenger signaling, will provide a systematic understanding of the STDP learning mechanism. Awareness of the dynamic cross-correlation principle will greatly facilitate the interpretation of data and may lead to the discovery of novel mechanisms involved in tuning the STDP curve of individual synapses within the network.

Furthermore, it has been shown that the self-organizing nature of stable cortical networks itself requires fluctuating synaptic strengths which results in a purely additive STDP mechanism. This is based on observed patterns of synaptic strength in the dynamics of dendritic spines of the rat hippocampus (Zheng et al., 2013). The robustness imposed by the dynHebb rule allowing additive dynamics to form the stable STDP learning which will ensure a stable network due to fluctuations in the local synaptic dynamics supports this concept completely.

## 6. Future work

Firstly, using the dynHebb rule, further work in dendritic computation and interaction between neurons has become possible. We are particularly interested in the robustness of developing neural networks (van Pelt et al., 2005). The stability introduced in the network due to the causal learning rule ensures that developing networks remain stable, even in the presence of large amount of input and network noise. We will show that for small networks, due to computational limitations, the dynHebb model will result in stable networks that have similar properties as observed experimentally, and are capable of self-calibration of their relative weights when connectivity changes over time. Secondly, the dynHebb model is purely phenomenological, which then begs the question how the mechanism of Hebbian learning is functionally employed in the biophysical synapse. The model does not preclude the elaboration of specific mechanisms, such as calcium signaling, or other relevant mechanisms. Indeed, one proposal would be to replace the dendritic cross-correlation term with one based on calcium waves, or to replace the depressing cross-correlation term with biophysical interpretations. Lastly, it would be extremely interesting to test the predictions and results of the dynHebb model in an experimental setup. We consider the continuous stimulation method to be very useful for the experimenter to provide a more robust framework to determine STDP. At the moment, we are exploring different avenues that would allow us to do this in the near future. By publicly demonstrating the capabilities of the dynHebb model, we envision an invigorating discussion on the relative merits of models vs. experimental results, ensuring that potential viewpoints from both experimental and theoretical understanding are valued to further our understanding of neural computation.

## Author contributions

Model design, implementation and analysis were performed by TVoS. RM, and AvO were involved in the design and interpretation of the results. All authors were involved in improving the manuscript.

### Conflict of interest statement

The authors declare that the research was conducted in the absence of any commercial or financial relationships that could be construed as a potential conflict of interest.
